# *Prevotella* and succinate treatments altered gut microbiota, increased laying performance, and suppressed hepatic lipid accumulation in laying hens

**DOI:** 10.1186/s40104-023-00975-5

**Published:** 2024-02-18

**Authors:** Min Liu, Zeyue Kang, Xikang Cao, Hongchao Jiao, Xiaojuan Wang, Jingpeng Zhao, Hai Lin

**Affiliations:** College of Animal Science and Technology, Shandong Provincial Key Laboratory of Animal Biotechnology and Disease Control and Prevention, Key Laboratory of Efficient Utilization of Non-Grain Feed Resources (Co-Construction by Ministry and Province), Ministry of Agriculture and Rural Affairs, Shandong Agricultural University, Tai’an, 271018 China

**Keywords:** Fatty liver, Gut microbiota, Laying hen, *Prevotella*, Succinate

## Abstract

**Background:**

This work aimed to investigate the potential benefits of administering *Prevotella* and its primary metabolite succinate on performance, hepatic lipid accumulation and gut microbiota in laying hens.

**Results:**

One hundred and fifty 58-week-old Hyline Brown laying hens, with laying rate below 80% and plasma triglyceride (TG) exceeding 5 mmol/L, were used in this study. The hens were randomly allocated into 5 groups and subjected to one of the following treatments: fed with a basal diet (negative control, NC), oral gavage of 3 mL/hen saline every other day (positive control, PC), gavage of 3 mL/hen *Prevotella melaninogenica* (10^7^ CFU/mL, PM) or 3 mL/hen *Prevotella copri* (10^7^ CFU/mL, *P. copri*) every other day, and basal diet supplemented with 0.25% sodium succinate (Succinate). The results showed that PM and *P. copri* treatments significantly improved laying rate compared to the PC (*P* < 0.05). The amount of lipid droplet was notably decreased by PM, *P. copri*, and Succinate treatments at week 4 and decreased by *P. copri* at week 8 (*P* < 0.05). Correspondingly, the plasma TG level in Succinate group was lower than that of PC (*P* < 0.05). Hepatic TG content, however, was not significantly influenced at week 4 and 8 (*P* > 0.05). PM treatment increased (*P* < 0.05) the mRNA levels of genes *PGC-1β* and *APB-5B* at week 4, and *ACC* and *CPT-1* at week 8. The results indicated enhanced antioxidant activities at week 8, as evidenced by reduced hepatic malondialdehyde (MDA) level and improved antioxidant enzymes activities in PM and Succinate groups (*P* < 0.05). Supplementing with *Prevotella* or succinate can alter the cecal microbiota. Specifically, the abundance of *Prevotella* in the Succinate group was significantly higher than that in the other 4 groups at the family and genus levels (*P* < 0.05).

**Conclusions:**

Oral intake of *Prevotella* and dietary supplementation of succinate can ameliorate lipid metabolism of laying hens. The beneficial effect of *Prevotella* is consistent across different species. The finding highlights that succinate, the primary metabolite of *Prevotella*, represents a more feasible feed additive for alleviating fatty liver in laying hens.

**Supplementary Information:**

The online version contains supplementary material available at 10.1186/s40104-023-00975-5.

## Background

The disorder of lipid metabolism is a prevalent symptom observed in the late stage of laying hens, which results in excessive lipid accumulation in the liver (fatty liver disease, FLD) and in turn decreased laying performance and increased mortality [1–5]. The fatty liver of laying hens is characterized by higher rates of hepatic lipogenesis and overaccumulation of triglycerides (TG) [6, 7].

Previous researches have provided evidence indicating that gut microbiota play a crucial role in the development of FLD [8, 9]. Recently, emerging evidence has highlighted the significant association between the gut-liver axis and the pathogenesis of liver injury in laying hens with fatty liver [10]. Dietary modulation has emerged as a potential therapeutic approach to address excess fat deposition in the liver, as it has the ability to modify the composition and metabolic activity of the gut microbiota [2, 11]. For example, Lee et al. [12] found that *Lactobacillus* and *Pediococcus* were a promising therapeutic approach in non-alcoholic fatty liver disease (NAFLD) by modulating gut-microbiome and inflammatory pathway in mammals. In recent years, there are increasing reports on the positive regulatory effects of bacteria and probiotics on fatty liver in monogastric animals [13, 14]. The intestinal dysbiosis is associated with the activation of the inflammatory cascade in the development of NAFLD [15]. The application of prebiotics and probiotics in laying hens could reshape the gut microbiota. For example, utilization of *Clostridium butyricum* and *Bacillus subtilis* enhanced the abundance of Firmicutes phylum in laying hens [16]. The supplementation of *C. butyricum* accelerated hepatic fatty acid oxidation, and shaped gut microbiota and bile acid (BA) profile, thus reducing fat deposition in the liver of aged laying hens [17]. Furthermore, mounting evidences support that gut microbiomes are involved in the regulation of hepatic metabolism of host via their metabolites short-chain fatty acids (SCFAs) [18, 19]. For example, gut *Akkermansia muciniphila* could ameliorate metabolic dysfunction-associated FLD by regulating lipid oxidation and improving gut-liver interactions through the modulation of L-aspartate metabolism [20]. *Lactobacillus salivarius* SNK-6 shown efficacy in inhibiting fat deposition and decreasing serum TG levels in a NAFLD model of laying hens, in which, SCFAs (acetate, butyrate, and propionate), the microbial metabolites, play an important role [21]. The positive effects of probiotics (*L. salivarius* and *B. subtilis*) on the host metabolism could improve the production performance and egg quality [22]. Dietary supplementation with *C. butyricum* showed beneficial with respect to hen performance and egg quality coordinate improving the gut health [23].

Dietary interventions are the potential effective approach for regulating the composition of gut microbiome and their associated metabolites. High dietary fiber intake leads to an increase in *Prevotella*, a genus of Gram-negative anaerobic bacteria [24, 25]. In addition, multiple studies have shown the association of *Prevotella* with glucose homeostasis [26–28]. However, there is limited knowledge about the role of *Prevotella* in the regulation of lipid metabolism. Recent studies indicated that bacteria regulate host metabolism through producing microbial metabolites [19, 29]. *Prevotella* has been reported to produce succinic acid, which has the potential to exert metabolic effects and overcome the challenges of anaerobic fermentation [27]. However, little is known about the function of *Prevotella* in the regulatory of lipid metabolism.

Recent evidences in mammals commended that the NAFLD development could be closely associated with mitochondrial dysfunction in the liver [30–32]. Mitochondrial function is related to the β-oxidation of fatty acids, TG synthesis, and the reactive oxygen species (ROS) accumulation. However, further elucidation is required to understand the mitochondrial function in the liver of FLD hens.

Therefore, we hypothesized that *Prevotella* and its metabolite, succinate, could alter lipid accumulation in the liver and improve laying performance of laying hens. The incidence of fatty liver adversely affects both laying performance and egg quality [10, 27, 28]. In the present study, the laying hens with a laying rate less than 80% and plasma TG levels exceeding 5 mmol/L were selected according to our preliminary clinical investigation. The effects of oral intake of *Prevotella* and dietary supplementation of succinate on laying performance and liver lipid metabolism of laying hens were measured.

## Methods

### Microorganisms culture

This work employed two distinct strains belonging to the *Prevotella* genus: *Prevotella melaninogenica* (ATCC 43982, ATCC, MD, USA) and *Prevotella copri* (BNCC 337399, BeNa Culture Collection, Beijing, China). The strains were precultured in complex KVLB broth medium (HB8817, Qingdao Hope Bio-Technology Co., Ltd., Qingdao, China) containing defibrated sheep blood (5%) (1001339-1, Qingdao Hope Bio-Technology Co., Ltd.), vitamin K_1_ (1‰) (2100501, Qingdao Hope Bio-Technology Co., Ltd.), and kanamycin-vancomycin complex (2‰) (HB8817a, Qingdao Hope Bio-Technology Co., Ltd.). The medium was cultured under the condition of 37 ºC in anaerobic environment.

### Birds and experimental design

A total of 150 healthy 58-week-old Hyline brown laying hens with similar laying rate below 80% (75.89% ± 1.47%) and similar plasma TG levels (Additional file 1) were randomly divided into 5 groups with 5 replicates per group and 6 hens per replicate and subjected to one of the following treatments: fed with the basal diet (negative control, NC), fed with the basal diet and oral gavage of 3 mL/hen saline every other day (positive control, PC), fed the basal diet and oral gavage of 3 mL/hen *Prevotella melaninogenica* (10^7^ CFU/mL, PM) or 3 mL/hen *Prevotella copri* (10^7^ CFU/mL, *P. copri*) every other day, and fed with the basal diet supplemented with 0.25% sodium succinate (S112882, Aladdin, Shanghai, China) and oral gavage of 3 mL saline/hen every other day (Succinate). The composition and nutrient levels of basal diet were presented in Table 1. The experiment lasted for 8 weeks and the hens had free access to feed and water during the whole experimental period. The lighting program was 16 h light period and 8 h dark period. Egg numbers and egg weight were recorded daily, laying rate and feed efficiency were calculated weekly.

**Table 1:** Composition and nutrient levels of experimental diets of laying hens

Ingredient, %	Content	Calculated nutrition levels, %	Content
Corn (8.5%)	58.41	Crude protein	16.50
Wheat bran	5.91	Metabolizable energy, kcal/kg	2,700
Soya-bean oil	1	Calcium	3.50
Soybean meal (46% CP)	22.68	Available phosphorus	0.404
Salt	0.35	Lysine	0.78
Limestone	9.61	Methionine	0.40
CaHPO_4_	1.44	Threonine	0.505
Choline chloride, 50%	0.09	Tryptophan	0.161
DL-Methionine, 99%	0.16	Methionine + cystine	0.619
Lysine, 99%	0.10		
Vitamin premix^a^	0.05		
Mineral premix^b^	0.20		

^a^Vitamin premix provides the following per kg of diet: VA, 8,800 IU; VD_3_, 3,300 IU; VE, 16.5 IU; VK, 2.2 mg; VB_1_, 1.7 mg; VB_2_, 5.5 mg; VB_3_, 6.6 mg; VB_5_, 28 mg; VB_6_, 3.3 mg; VB_7_, 0.1 mg; VB_9_, 0.6 mg; VB_12_, 0.05 mg

^b^Mineral premix provides the following per kg of diet: Fe (as ferrous sulfate), 55 mg; Zn (as zinc sulfate), 88 mg; Mn (as manganese sulfate), 88 mg; Cu (as copper sulfate) 5.5 mg; I (as potassium iodide), 1.7 mg; Se (as sodium selenite), 0.3 mg

### Sample collection

Blood samples were collected every two weeks. One hen was randomly selected from each replicate in every treatment, a blood sample was collected from the brachial vein of each bird and centrifuged at 4 ºC to get plasma and stored at −80 ºC for further analysis. Five eggs were respectively obtained from each replicate at week 4 and week 8 and used for the measurement of egg quality. At the end of week 4 and 8, eight hens were randomly obtained from each replicate. The hens were weighted and sacrificed by exsanguination after cervical dislocation [33, 34]. The liver was excised and weighted, and then the morphological observation was conducted. After removed blood with PBS, tissue sample was obtained from the middle part of the liver, part of the sample was fixed in 4% formaldehyde for morphology observation, and another part of sample was snap frozen in liquid nitrogen for further analysis. The abdominal fat pad was weight to calculated organ index. The cecal content were obtained and was immediately snap frozen in liquid nitrogen and stored at −80 °C for further analysis.

### Physical egg parameters

Eggshell breaking strength was evaluated using the Egg Force Reader (EFG-0503, Robotmation Co., Ltd., Tokyo, Japan), and sharp end, equator, blunt end was measured to obtain the value of eggshell thickness with the Egg Shell Thickness Gauge (ESTG-1, ORKA Food Technology LLC, West Bountiful, UT, USA). Albumen height, Haugh units, and yolk color were tested by the Egg Multi Tester EMT-500 (EMT-5200, Robotmation Co., Ltd., Tokyo, Japan).

### Plasma parameters

The plasma TG, total cholesterol (TCH), glucose (GLU), low density lipoprotein cholesterol (LDL-C), high density lipoprotein cholesterol (HDL-C) levels were analyzed using the Hitachi L-7020 fully automatic biochemical analyzer (7020, HITAGHUI, Tokyo, Japan).

### Hepatic TG and TCH content measurements

TG and TCH contents in the liver were measured using commercial kits (A110-1-1 and A111-1-1, GPO-PAP and CHOD-PAP, Nanjing Jiancheng Biotechnology Institute, Nanjing, China).

### Liver histological observation

The liver samples were cut into 5 μm serial sections sliced, Hematoxylin and eosin (H&E) strained, and sheet sealed, each slice was chosen with 10 visions for observing the liver morphology using a light microscope (CK-40, Olympus, Tokyo, Japan).

### Oil Red O staining

Liver sample was put in sucrose solution (15%) for 6 h and then shifted in sucrose solution (30%) overnight at 4 ºC. The tissue was embedded in OTC and immediately put on the −20 ºC quick-freezing machine, and then cut into 8 μm slide by the CryoStar NX50 HOVPD freezing slicer (CryoStar NX50 HOVPD, Thermo, MA, USA). These sliced stored at −20 ºC. The room temperature was restored for 20 min before staining. It was stained for 30 min in a fresh diluted Oil Red O (ORO) solution (G1262, Solarbio, Beijing, China). After color separation of 60% isopropanol (20 s), softly flow flush of H_2_O (3 min), lining dyeing of hematoxylin (20 s), rinsing with distilled water, the slide was mounted in glycerine jelly. The image of each group was photographed with a light microscope (CK-40, Olympus, Tokyo, Japan). The lipid droplets in ORO staining was measured by Image J software and the ratio of lipid droplets in ORO staining was calculated.

### Lipid metabolism associated enzyme activities and free fat acid content

The hepatic fatty acid synthase (FAS) (H231-1-1, Nanjing Jiancheng Biotechnology Institute, Nanjing, China), lipoprotein lipase (LPL), hepatic lipase (HL) activities and free fat acid (FFA) contents (ml060836, ml036976, and ml556655, Shanghai Enzyme-linked Biotechnology Co., Ltd., Shanghai, China) and malic enzyme (ME) (BC1120, Solarbio, Beijing, China) were analyzed using commercial kits.

### Hepatic antioxidant enzyme activities

The activities of glutathione (GSH), glutathione peroxidase (GSH-Px), catalase (CAT), superoxide dismutase (SOD), and contents of malondialdehyde (MDA) and total antioxidant capacity (T-AOC) were performed by commercial kits (A006-2-1, A005-1-2, A007-1-1, A001-3-2, A003-1-2, A015-2-1, Nanjing Jiancheng Biotechnology Institute, Nanjing, China).

### RNA extraction, cDNA synthesis, and quantitative real-time PCR

The method of RNA isolation and reverse transcription and qRT-PCR was followed. Total RNA of the liver was isolated using Trizol reagent (M5100, New Cell & Molecular Biotech Co., Ltd., Suzhou, China). The concentration of RNA was texted by Nanodrop 2000c spectrophotometer (Thermo, MA, USA). The mRNA was reversed transcribed into cDNA by Primer Script RT reagent kit (CW2569, Jiangsu CoWin Biotech, Taizhou, China). Gene expression of the liver was measured by quantitative real-time PCR (7500, ABI, CA, USA), including acetyl-CoA carboxylase (*ACC*), adenosine monophosphate-5B (*ATP-5B*), cytochrome IV (*COX IV*), carnitine palmitoyltransferase 1 (*CPT-1*), *FAS*, isocitrate dehydrogenase 3α (*IDH3α*), nuclear respiratory factor 1 (*NRF1*), peroxisome proliferator-activated receptor γ-1α (*PGC-1α*), *PGC-1β*, peroxisome proliferator-activated receptor γ (*PPARγ*), sterol regulatory element binding proteins-1c (*SREBP-1c*), mitochondrial transcription factor A (*TFAM*). The primer was synthesized by Sangon Biotech (Shanghai) Co., Ltd. (Table 2), and *GAPDH* was as the housekeeping gene and calculated using the 2^−ΔΔCt^ method.

**Table 2:** Primers used in the work

Gene	Primer sequence (5´→3´)	Accession No.
*GAPDH*	F: ACATGGCATCCAAGGAGTGAG R: GGGGAGACAGAAGGGAACAGA	NM-204305.2
*ACC*	F: AATTGGCAGCTTTGGAGGTGT R: TCTGTTTGGGTGGGAGGTG	NM-205505
*FAS*	F: CTATCGACACAGCCTGCTCCT R: CAGAATGTTGACCCCTCCTACC	J03860
*PPARγ*	F: CCAGCGACATCGACCAGTT R: GGTGATTTGTCTGTCGTGTTTCCC	AF163811
*CPT1*	F: GGGACCTGAAACCAGAGAACG R: ACAGAGGAGGGCATAGAGGATG	AY675193
*SREBP1c*	F: GCCCTCTGTGCCTTTGTCTTC R: ACTCAGCCATGATGCTTCTTCC	NM_204126.2
*PGC-1α*	F: GACTCAGGTGTCAATGGAAGTG R: ATCAGAACAAGCCCTGTGGT	NC_006091
*PGC-1β*	F: AGGGTCCTCGACACGCACTA R: CTTTGTTAAACTGGCTCTGATCTCC	NC_006100.5
*ATP-5B*	F: AGAGATGAGCGTCGAGCAGGAG R: ACACCAGCGAACACCGAATAACC	NC_003283.11
*NRF1*	F: GGCCAACGTCCGAAGTGAT R: CCATGACACCCGCTGCTT	NC_052532.1
*TFAM*	F: AGCAGAACCCAGAACTGAAC R: CAAGCACAGTCAATTCTCTC	NC_0.2537.1
*COX IV*	F: CAACCACCCAACACTCTGG R: CTCTTGCCTCCTCTTCCTCA	NM_001030577
*IDH3a*	F: TGCTGGATTGATTGGAGGTCTTGG R: AGGTGCTGTTCCATGAACCGATTC	NC_000015.10

### 16S rRNA sequencing analysis

DNA was extracted from cecal content using the Magnetic Soil and Stool DNA kit (DP336-02, TianGen, Beijing, China) and its integrity was tested through electrophoresis on a 1% agarose gel, and then its amplification was performed with the TransStart® FastPfu DNA Polymerase (AP221-01, TransGen Biotech, Beijing, China). The PCR production as the templates was amplified using the universal primer (338F: 5´-ACTCCTACGGGAGGCAGCAG-3´, 806R: 5´-GGACTACHVGGGTWTCTAAT-3´) targeting the V3-V4 hyper variable region. The PCR reaction conditions consisted of an initial denaturation step at 95 ºC for 3 min, followed by 30 cycles of denaturation at 95 ºC 30 s, annealing at 55 ºC for 30 s, and extension at 72 ºC for 30 s, with a final extension of 10 min at 72 ºC. The integrity was assessed by electrophoresis on a 2% agarose gel, and then purified by the AxyPrep DNA Gel extraction kit (AP-GX-50, Axygen Scientific, Inc., CA, USA). The purification was qualified and sequenced by the Quantus™ Fluorometer (E6150, Promega, Madison, WI, USA). Purified amplicons were pooled in equimolar and paired-end sequenced on an Illumina Novaseq 6000 PE250 platform (Illumina, San Diego, CA, USA). Raw data FASTQ files were imported by QIIME2 system. In our work, the average count of raw sequences and sample depth amounts to 86,561 bp. Demultiplexed sequences from each sample were quality filtered and trimmed, de-noised, and merged. Subsequently, chimeric sequences were identified and removed to obtain the feature table of amplicon sequence variant (ASV) and further generate the taxonomy table. The average sequence counts, post-quality control, is 67,199 bp. Subsequently, the QIIME2 feature-classifier plugin was used to align the ASV sequences with a pre-trained GREENGENES 13_8 99% database for the purpose of generating the taxonomy table. The linear discriminant analysis effect size (LEfSe) tool was utilized for detecting bacterial exhibiting differential abundance within the sample and groups at phylum level (LDA ≥ 4.0). Diversity metrics were calculated using the core-diversity plugin within QIIME2. Alpha diversity indices at the feature level, including observed operational taxonomic unit (OTU), the Chao1 estimator, Shannon index, Simpson index and Faith PD index, were computed to estimate microbial diversity within individual samples. Beta diversity distance measurements, Bray–Curtis, was employed to explore structural variations in microbial communities across the samples. The results were visualized using principal coordinate analysis (PCoA). Partial least squares discriminant analysis (PLS-DA) was employed as a supervised model for discerning microbiota variations among the groups. This analysis was conducted using the "plsda" function within the R package "mixOmics". Redundancy analysis (RDA) was conducted to elucidate the connections between microbial communities and environmental factors, utilizing relative abundances of microbial species at various taxonomic levels. This analysis was performed using the "vegan" package in R. Co-occurrence analysis was conducted by calculating Spearman's rank correlations among the dominant taxonomic groups, and the results were visualized using a network plot to represent the relationships among these taxa. The 16S rRNA sequencing of the gut microbiome was performed in Wekemo Tech Group Co., Ltd. (Shenzhen, China).

### Statistical analysis

All the data were presented as means ± SEM. For variables laying performance, plasma parameters, hepatic enzyme activity and redox parameters, and hepatic gene expression, the main effect of treatment was estimated with one-way ANOVA (SAS software, version 9.4). Means were compared using Tukey post-tests where significant (*P* < 0.05). Statistically significant was determined at a* P* value threshold of < 0.05.

For microbial community analysis, appropriate methods include ANCOM, ANOVA, Kruskal Wallis, and LEfSe were used to identify the bacteria with different abundance among samples and groups.

## Results

### *Prevotella* improved the laying performance in laying hens

The laying rate in the PM and *P. copri* groups was significantly higher than that in the PC group (*P* < 0.05, Table 3). *P. copri* treatment significantly increased (*P* < 0.05) egg weight in comparison to NC group. Moreover, *P. copri* treatment notably elevated egg mass when compared to PC and NC groups (*P* < 0.05). The ADFI and FCR were not altered (*P* > 0.05) by dietary treatment. For the egg quality, there was no difference (*P* > 0.05) in egg-shaped index, eggshell thickness, eggshell strength, albumen height, yolk color, Haugh units, eggshell proportion and yolk proportion among treatments at the fourth and eighth weeks (Additional file 2).

**Table 3:** Effect of *Prevotella* and sodium succinate on the laying performance in the laying hens

Item	NC	PC	PM	*P. copri*	Succinate	*P*-value
Laying rate, %	73.45 ± 1.25^ab^	71.33 ± 1.08^b^	76.66 ± 1.83^a^	78.25 ± 1.94^a^	75.31 ± 1.91^ab^	0.045
Egg weight, g	58.81 ± 0.93^bc^	60.37 ± 0.36^ab^	60.57 ± 0.64^ab^	61.26 ± 0.33^a^	58.33 ± 0.73^c^	0.019
Egg mass, g/hen/d	43.45 ± 0.75^bc^	42.28 ± 0.75^c^	46.23 ± 0.92^ab^	48.31 ± 1.57^a^	44.37 ± 1.37^bc^	0.005
ADFI, g/d/h	104.17 ± 2.91	107.47 ± 2.91	108.21 ± 2.83	105.86 ± 3.39	105.93 ± 3.82	0.951
FCR, g/g	2.42 ± 0.08	2.54 ± 0.06	2.34 ± 0.03	2.29 ± 0.05	2.40 ± 0.09	0.106

The assessment measures were conducted at week 8. All data are presented as mean ± SEM (*n* = 5). ^a−c^Means with different superscripts within a column differ significantly (*P* < 0.05)

### *Prevotella* and succinate decreases plasma TG levels

**Fig. 1:**
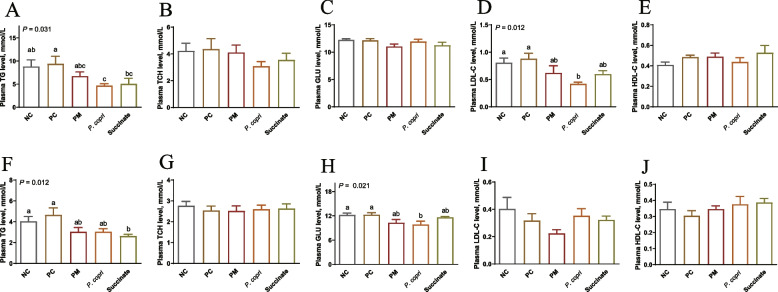
Effect of *Prevotella* (3 × 10^7^ CFU) and sodium succinate (0.25%) on plasma parameters of hens. **A** and **F** Triglyceride (TG). **B** and **G** Total cholesterol (TCH). **C** and **H** Glucose (GLU). **D** and **I** Low-density lipoprotein-cholesterol (LDL-C). **E** and **J** High-density lipoprotein-cholesterol (HDL-C). The assessment measures were conducted at two different time points: at week 4 (**A**–**E**) and week 8 (**F**–**J**). ^a−c^Means with different letter differ significantly (*P* < 0.05)

The initial TG, TCH, GLU, LDL-C and HDL-C content in plasma of laying hens among all the groups showed no remarkable difference (*P* > 0.05, Additional file 1). At the fourth week, plasma TG content was notable lowered (*P* < 0.05) in *P. copri* compared with NC and PC groups and was decreased by Succinate treatment compared to PC (*P* < 0.05, Fig. 1A). Plasma TCH, GLU, and HDL-C levels were not influenced by treatment (*P* > 0.05, Fig. 1B, C and E). In contrast, plasma LDL-C level was decreased by *P. copri* treatment (*P* < 0.05) compared with NC and PC groups (Fig. 1D). At week 8, however, plasma TG was decreased (*P* < 0.05) by Succinate treatment compared to NC and PC groups and there was no detectable difference among PM, Succinate, and *P. copri* treatments (*P* < 0.05, Fig. 1F). The GLU level in *P. copri* group was significantly lower than those in the NC and PC groups (*P* < 0.05, Fig. 1H). Dietary treatments had no significant effect on TCH, LDL-C, and HDL-C levels (*P* > 0.05, Fig. 1G, I, J).

### *Prevotella* and succinate suppress the hepatic TG accumulation

**Fig. 2:**
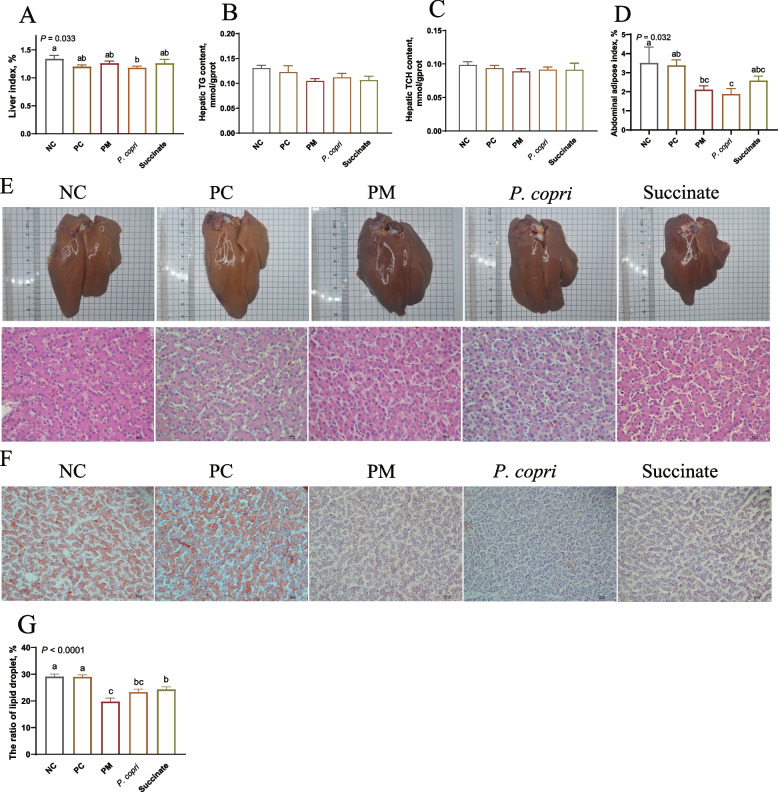
*Prevotella* (3 × 10^7^ CFU) and sodium succinate (0.25%) suppressed the hepatic lipid accumulation at week 4. **A** Liver index. **B** Hepatic triglyceride (TG) content. **C** Hepatic total cholesterol (TCH) content. **D** Abdominal fat pad index. **E** Liver morphology (upper), and H&E staining (lower). **F** Hepatic ORO staining. **G** The ratio of lipid droplets in ORO staining. ^a−c^Means with different letters differ significantly (*P* < 0.05)

**Fig. 3:**
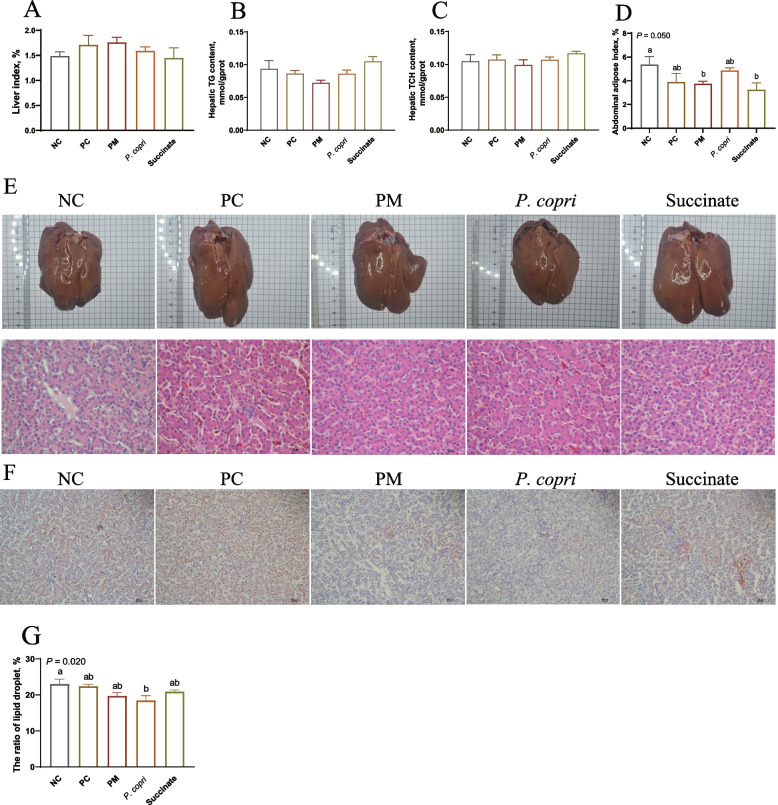
*Prevotella* (3 × 10^7^ CFU) and sodium succinate (0.25%) suppressed the hepatic lipid accumulation at week 8. **A** Liver index. **B** Hepatic triglyceride (TG) content. **C** Hepatic total cholesterol (TCH) content. **D** Abdominal fat pad index. **E** Liver morphology (upper) and H&E staining (lower). **F** Hepatic ORO staining. **G** The ratio of lipid droplets in ORO staining. ^a,b^Means with different letters differ significantly (*P* < 0.05)

At week 4, *P. copri* treatment decreased liver index compared to NC group (*P* < 0.05, Fig. 2A). All the treatments had no significant influence (*P* > 0.05) on hepatic TG and TCH contents (Fig. 2B and C). The abdominal fat pad index was decreased by *P. copri* compared to NC and PC groups, and decreased in PM group compared to NC group (*P* < 0.05, Fig. 2D). At week 8, PM and Succinate treatments notably decreased the abdominal fat pad index compared to NC group (*P* < 0.05, Fig. 3D). The liver of NC group and PC group appeared yellow color, while the liver of hens in PM, *P. copri*, and Succinate groups appeared normal red color (Fig. 2E and 3E). The ORO staining and H&E staining showed that there was less amount of lipid droplets in PM, *P. copri*, and Succinate treatments, compared with PC or NC group (Fig. 2E and F and 3E and F). Figure 2G revealed that the lipid droplets in the PM, *P. copri* and Succinate groups exhibited a significant decrease compared to both the NC and PC groups at the fourth week (*P* < 0.01). At week 8, liver index and hepatic TG and TCH contents were not changed (*P* > 0.05) by dietary treatment (Fig. 3A–C). The abdominal fat pad index was decreased (*P* = 0.05) by PM and Succinate treatments compared with NC (Fig. 3D). The *P. copri* treatment had less lipid droplets in comparison to the NC group (*P* < 0.05, Fig. 3G).

### The effect of *Prevotella* and succinate on lipid metabolism associated enzyme activities and FFA content

**Fig. 4:**
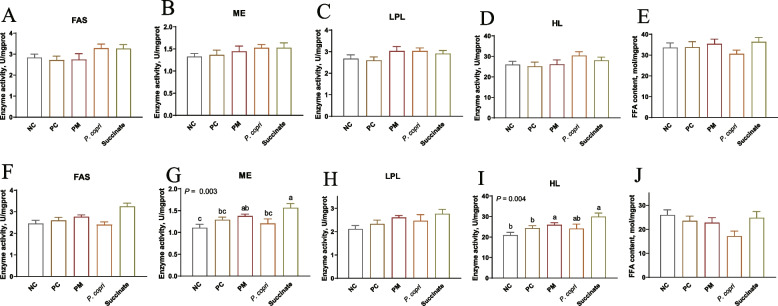
Effect of *Prevotella* and sodium succinate on enzymes activities related to hepatic lipid metabolism. **A** and **F** Fatty acid synthase (FAS) activity. **B** and **G** Malic enzyme (ME) activity. **C** and **H** Lipoprotein lipase (LPL) activity. **D** and **I** Hepatic lipase (HL) activity. **E** and **J** Free fat acid (FFA) content. The assessment measures were conducted at two different time points: at week 4 (**A**–**E**) and at week 8 (**F–J**). ^a−c^Means with different letters differ significantly (*P* < 0.05)

At week 4, FAS, ME, LPL, and HL activities and FFA content were not significant influenced by treatment (*P* > 0.05, Fig. 4A–E). At week 8, PM treatment increased (*P* < 0.05) ME activity compared with NC, while Succinate notably increased (*P* < 0.01) the activity of ME compared to NC, PC, and *P. copri* groups (Fig. 4G). The HL activity in the PM and Succinate groups exhibited a significant increase compared to NC and PC groups (*P* < 0.01, Fig. 4I). The FAS and LPL activities and FFA level were not altered (*P* > 0.05) by treatment (Fig. 4F, H and J).

### *Prevotella* and succinate regulate CPT-1 and mitochondrial function related mRNA expression

**Fig. 5:**
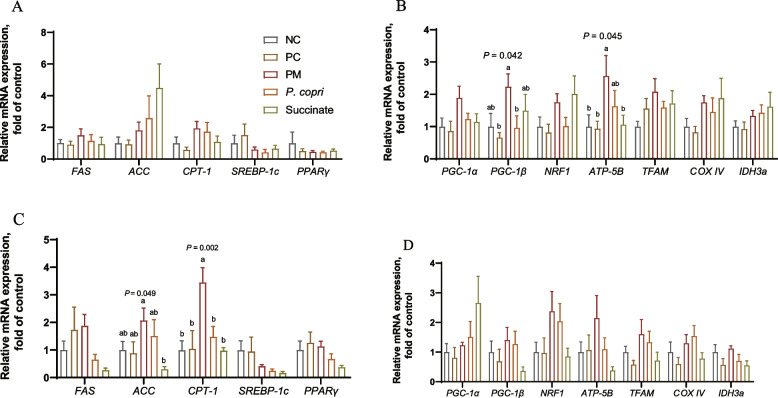
*Prevotella* and sodium succinate regulated hepatic lipid metabolism and mitochondrial function related mRNA expression. The assessment measures were conducted at two different time points: at week 4 (**A** and **B**) and at week 8 (**C** and **D**). ^a−b^Means with different letters differ significantly (*P* < 0.05)

At week 4, for the key genes related to lipid metabolism, the mRNA expression of *FAS*, *ACC*, *CPT-1*, *SREBP-1c*, and *PPARγ* displayed no obvious difference among these five groups (*P* > 0.05, Fig. 5A). The genes expression associated with mitochondrial function was then evaluated. The mRNA expression of *PGC-1β* was increased (*P* < 0.05) by PM compared with PC and *P. copri* (Fig. 5B). In contrast, the expression of *ATP-5B* was significantly increased in PM group compared with PC, NC, and Succinate (*P* < 0.05, Fig. 5B). The expression of *PGC-1α*, *NRF1*, *TFAM*, *COX IV*, and *IDH3a* was not changed by all the treatment (*P* > 0.05, Fig. 5B).

At week 8, the expression level of *ACC* was significantly reduced in Succinate group when compared with PM group (*P* < 0.05, Fig. 5C). The *CPT-1* expression level was increased by PM compared to the other four groups (*P* < 0.01, Fig. 5C). By contrary, the expression of *FAS*, *SREBP-1c*, and *PPARγ* was not changed by dietary treatment (*P* > 0.05, Fig. 5C). The expression of genes *PGC-1α*, *PGC-1β*, *NRF1*, *ATP-5B*, *TFAM*, *COX IV*, and *IDH3a* was not changed by treatment (*P* > 0.05, Fig. 5D).

### *Prevotella* and succinate could improve the hepatic antioxidant activities

**Fig. 6:**
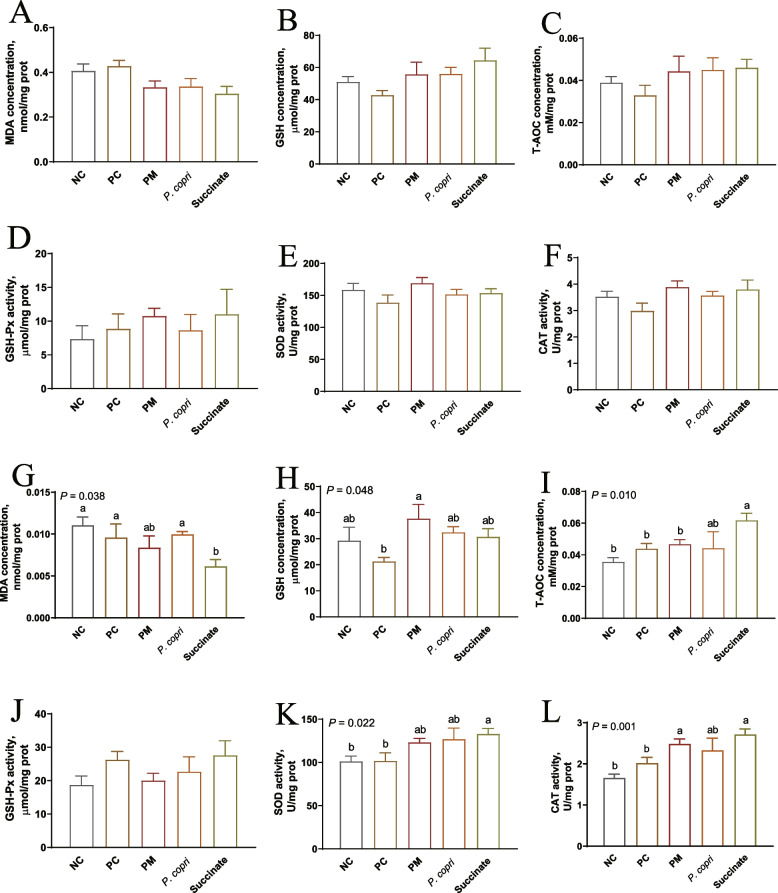
*Prevotella* and sodium succinate improved the hepatic antioxidant activities. **A** and **G** Malondialdehyde (MDA) content. **B** and **H** Glutathione (GSH) concentration. **C** and **I** Total antioxidant capacity (T-AOC) concentration. **D** and **J** Glutathione peroxidase (GSH-Px) activity. **E** and **K** Superoxide dismutase (SOD) activity. **F** and **L** Catalase (CAT) activity. The assessment measures were conducted at two different time points: at week 4 (**A**–**F**) and at week 8 (**G**–**L**). ^a,b^Means with different letters differ significantly (*P* < 0.05)

At week 4, all the measured hepatic oxidative parameters MDA, GSH, T-AOC, GSH-Px, SOD, and CAT were not significantly (*P* > 0.05) changed by treatment (Fig. 6A–F). At week 8, MDA levels in the Succinate group were significantly lower than those in the NC, PC, and *P*. *copri* groups (*P* < 0.05, Fig. 6G). PM treatment significantly increased the levels of GSH compared to PC group (*P* < 0.05, Fig. 6H). The T-AOC content was significantly (*P* < 0.05) increased by Succinate treatment, compared with PC, NC, and PM (Fig. 6I). Compared with PC and NC groups, Succinate had higher (*P* < 0.05) SOD activity, while PM and Succinate treatments had elevated (*P* < 0.01) CAT activity (Fig. 6K and L). The GSH-Px activity was not changed by treatment (*P* > 0.05, Fig. 6J).

### Intestinal microbial diversity and community

**Fig. 7:**
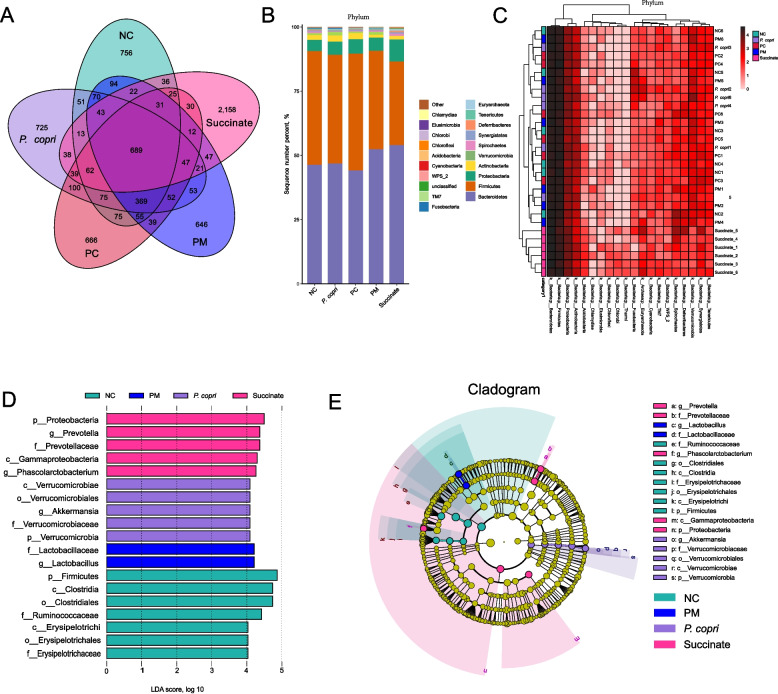
Effect of *Prevotella* and sodium succinate on the gut microbiota. **A** Venn diagram illustrated the overlap of operational taxonomic unit (OTU) in all tested chicken. **B** The composition of gut microbiota of community bar plot analysis on phylum level. **C** Heatmap hierarchical cluster analysis based on the 20 most abundant phylum among groups. **D** Linear discriminant analysis effect size (LEfSe) analyses (LDA score ≥ 4.0) at the phylum level to the genus level. **E** Cladogram generated from LEfSe analysis (LDA score ≥ 4.0). Different color nodes represent the significant enriched microbial groups in the corresponding treatment groups which have a significant impact on the differences among groups, and the diameters of the circles are proportional to the axon’s abundance

As shown in Fig. 7A, the *Prevotella* and succinate could alter the abundance of microbiome, particularly by enriching the abundance of microbiota diversity through the presence of succinate. Phylum distributions and heatmap analysis revealed changes in composition and clustering due to the treatment of *Prevotella* and succinate, particularly in the Succinate group (Fig. 7B and C). The LEfSe analysis (LDA > 4.0) indicated significant differences in the abundance of OTU at the phylum levels within the entire microbiota (Fig. 7D and E). In the *P. copri* group, LEfSe highlighted the greater differential abundance of Verrucomicrobia (phylum level) and its derivatives (Verrucomicrobiae, Verrucomicrobiales, and Verrucomicrobiaceae), and the genus *Akkermansia*. The marker microorganism in the Succinate group belonged to the phylum Proteobacteria, class Gammaproteobacteria, genus *Phascolarctobacterium*, family Prevotellaceae, and its derivative genus *Prevotella*.

**Fig. 8:**
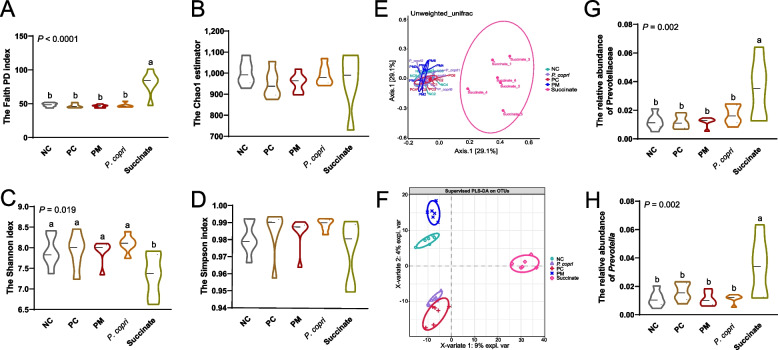
Effect of *Prevotella* and sodium succinate on the gut microbiota. **A–D** Alpha diversity among groups (Faith PD, Chao1, Shannon, and Simpson index). **E** Principal coordinate analysis (PCoA) based on Bray–Curtis dissimilarity. **F** Partial least squares discriminant analysis (PLS-DA). **G** The relative abundance of Prevotellaceae. **H** The relative abundance of *Prevotella*. ^a,b^Means with different letters differ significantly (*P* < 0.05)

Faith PD index was increased significantly by the supplementation of succinate compared with NC, PC, PM, and *P. copri* groups (*P* < 0.01, Fig. 8A), indicating a notable increase in evolutionary divergence and microbiome diversity. There were no significant differences in the alpha diversity of the gut microbiome between the *Prevotella* (PM or *P. copri*) group and control (NC or PC) group (*P* > 0.05, Fig. 8A–D). However, the Succinate group exhibited a decreased Shannon index compared to NC, PC, PM, and *P. copri* groups (*P* < 0.05, Fig. 8C). The PCoA using the Bray distance metric demonstrated distinct separation between the succinate dietary group and other four groups, including NC, PC, PM and *P. copri* groups (Fig. 8E). Additionally, PLS-DA clearly demonstrated distinct classification separation among the NC, PC, PM, *P. copri* and Succinate groups (Fig. 8F). The relative abundance of Prevotellaceae and *Prevotella* remarkably increased in the Succinate group compared to NC, PC, PM, and *P. copri* groups (*P* < 0.01, Fig. 8G and H).

### Association analysis and phylogenetics analysis

**Fig. 9:**
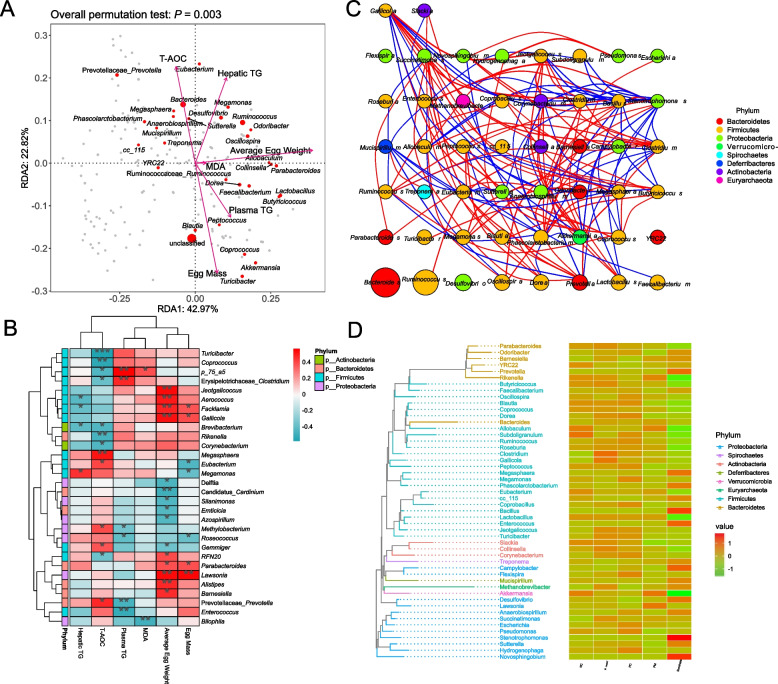
*Prevotella* and sodium succinate influenced the correlations between intestinal flora and physicochemical parameters. **A** Redundancy analysis (RDA), parameters are represented by arrows. The longer the arrows are, the greater the correlation coefficients are. The Angle between the arrow line and the ordering axis represents the correlation between an environmental factor and the ordering axis. The smaller the Angle is, the higher the correlation will be. The larger the dot, the higher the corresponding species abundance. **B** Spearman's correlation analysis between serum biochemical indexes and cecal microbiota at the phylum level, R-values are shown in different colors in the heatmap, ^*^P < 0.05, ^**^P < 0.01, ^***^P < 0.001. **C** The genus-level microbiome Spearman’s correlation network, the circles represent a species, the size represents its relative abundance, and different colors represent different phylum species classification. The lines between the circles represent significant correlation between the two species (*P* < 0.05), and the lines color red represents positive correlation and blue represents negative correlation. The thicker the lines, the greater the absolute value of the correlation coefficient. **D** Phylogenetic tree and heatmap, on the left, different colored branches of the evolutionary tree represent different phyla, and each end branch represents an OTU, annotated with the genus classification to which the corresponding OTU belongs, the heat map on the right represents the normalized abundance, with larger values representing higher relative abundance

RDA revealed that hepatic TG levels were among the most influential parameters in explaining the majority of variations in the community compositions of the samples (Fig. 9A). It indicated a negative correlation between the relative abundance of *Prevotella* and plasma TG levels, while a positive correlation was observed between the relative abundance of *Prevotella* and T-AOC levels. The correlation heatmap revealed a significant negative correlation between plasma TG levels and the abundance of *Prevotella* (*P* < 0.01). Additionally, a noteworthy positive correlation was observed between T-AOC levels and the relative abundance of *Prevotella* (*P* < 0.05) (Fig. 9B). The genus-level microbiome Spearman's correlation network, as depicted in Fig. 9C, revealed intricate interactions among the microbial species present in the samples. In terms of the relative abundance of *Prevotella*, it exhibited a direct positive correlation with *Dorea*, *Akkermansia*, *Phascolarctobacterium*, *Blautia*, *Sutterella*, *Eubacterium*, and a direct negative correlation with *Mucispirillum* and *Clostridium*. The phylogenetic tree of intestinal microbes showed that *Prevotella*, *YRC22*, *Barnesiella*, *Odoribacter*, *Parabacteroides*, and *Rikenella* clustered together in the same phylogenetic clades, and they belonged to the phylum Bacteroidetes, and *Prevotella* was particularly enriched in the Succinate group.

## Discussion

The incidence of fatty liver increases with laying hens adversely affects both laying performance and egg quality, resulting in economic losses [10, 27, 28]. In this study, the laying hens with relative lower laying rate (< 80%) and higher plasma TG level (> 5 mmol/L) were selected. The result indicated that *Prevotella*, along with its associated metabolite succinate, effectively improved the laying performance of laying hens by alleviating hepatic lipid accumulation. Oral administration of *Prevotella* significantly reduce the plasma TG level. Succinate, the main metabolite from *Prevotella* [35], can play the alternative role in reducing the hepatic lipid droplet by improving mitochondrial function. Additionally, succinate eliminated the challenges with anaerobic culture. This work demonstrated that succinate could be considered as a potential feed additive for enhancing the laying performance and alleviating fatty liver and of laying hens.

Gut microbiome is associated with laying performance of hens [10, 36, 37]. Zhang et al. [22] demonstrated that the application of probiotics, specifically a combination of heat-inactivated *L. salivarius*^CB^ and *B. subtilis*, led to significant increases in egg production and daily egg yield, as well as significant reductions in feed conversion. In this study, the increased egg mass by *P. copri* treatment compared with PC and NC indicated the beneficial effect of *P. copri* treatment, in line with findings in previous probiotic research [22, 38]. The improved laying rate and egg mass by PM compared to PC indicating the favorable effect of PM treatment on laying performance. Moreover, the comparable effect of PM with *P. copri* suggests that the positive effect of *Prevotella* on laying performance is consistent across different species. Moreover, the laying performance was not significantly altered in Succinate group, suggesting the metabolites of PM and *P. copri* play a minor role in the supplemental effect of *Prevotella* on laying performance. Feed intake was not changed by dietary treatment, indicating that *Prevotella* or succinate supplementation has no unfavorable influence on the appetite. In the present study, the supplemental effect of succinate on laying performance was less obvious compared with PM or *P. copri*. The adequate supplemental dose needs further determined.

In previous studies, it is observed that the introduction of probiotic strains like *C. butyricum* could increase eggshell strength [23], and *B. subtilis* was associated with improvements in albumen height and Haugh unit [39]. In this study, however, all the measured parameters related egg quality were not influenced by dietary treatments. If the effect of probiotic strain and their metabolites on egg quality needs to be investigated further.

Liver is the main site for lipid synthesis in poultry, and the occurrence of fatty liver is strongly associated with blood lipid disorders [40–42]. Our work found that none of the three treatments PM, *P. copri*, or Succinate had any detectable influence on the HDL-C level, which aligns with the findings of Wu et al. [43]. Wu et al. [43] reported that the lack of significant changes in HDL-C may be attributed to liver damage without hepatic lipidosis, and the limited diagnostic value of liver function and blood lipid metabolism biomarkers for NAFLD. In the present work, the supplement of *Prevotella* and succinate decrease of plasma TG levels and plasma LDL-C levels. The findings are consistent with a previous study by Zhu et al. [44], which demonstrated that dietary supplementation with an herbaceous mixture could effectively lower serum TG and LDL-C levels. *Prevotella* was shown to effectively reduce TG levels in patients with hyperlipidemia or cardiovascular disease and prevent liver disorders [45]. Elevated TG levels are widely established as the primary characteristic of NAFLD, leading to poor egg production, high mortality and lower production performance in layer chickens [46]. And in our work, we observed a significant improvement in the production performance of laying hens with reduced levels of TG. However, another study by Yao et al. [47] demonstrated that dimethylglycine supplementation could improve the laying rate by reducing of the abdominal fat rate, without affecting plasma TG levels.

Excessive accumulation of TGs can be observed through anatomical and pathological features, including liver enlargement, brittleness, and a tawny color [48]. TG is the main lipid deposited in the liver [49]. The histological changes observed in the livers of these hens provide evidence for anatomical pathological features. The hepatocyte exhibited similarities to those previously described for NAFLD [50]. Administration of PM or succinate can induce alterations in the pathological changes, thereby indicating a potential alleviation of the TGs accumulation in the liver of laying hens. The decreased abdominal fat accumulation by Succinate was in line with the reduced plasma TG level. The decreased plasma TG level, lipid droplets in hepatocytes, and abdominal fat pad index in PM, *P. copri* or Succinate treatment indicated the arrested development of fatty liver, align with previous works [40, 51], in which the increased lipid droplets and fat vacuoles accumulation were observed in the livers of fatty liver-afflicted laying hens. And these syndromes could be alleviated by dietary probiotics, such as *Bacillus* [38], *L. salivarius* [21], *A. muciniphila* [52], and *C. butyricum* [53].

The de novo hepatic lipogenesis is strongly associated with the functioning of hepatic mitochondria, which is the important contributing factor to NAFLD development [54]. The *ACC* is the rate-limiting enzyme in fatty acid synthesis and is believed to be responsible for inhibiting fat deposition [55]. Another fatty acid de novo synthesis enzyme, *FAS*, resides predominantly in the liver and fat tissues, where it facilitates the conversion of acetyl-CoA and malonyl-CoA into long-chain fatty acids [56]. Moreover, down-regulation of the transcription factor SREBP-1c, which binds to the DNA sequence involved in sterol biosynthesis, has been suggested as the cause for the decreased synthesis of sterols and TC. We found that PM or Succinate increased ME and HL activities, while had no obvious influence on the enzyme activities and gene expression related to lipid biosynthesis, suggesting the lipid synthesis play a minor role in the reduced hepatocytic lipid droplet by PM and succinate. The elevated expression of *CPT-1*, a rate-limiting enzyme in mitochondrial* β*-oxidation, indicated that PM promoted the process of fatty acid *β*-oxidation. This speculation was supported by the elevated expression of genes related to mitochondrial function. These results are in accordance with previous studies that have reported the prevention of downregulation of fatty acid *β*-oxidation genes by betaine further, thereby alleviating hepatic lipid accumulation [57]. This result suggested that PM or succinate has a beneficial effect on mitochondrial function.

Mitochondrial dysfunction can lead to electron leakage at respiratory complexes, resulting in the excess accumulation of ROS. And excessive ROS can disrupt the balance between oxidation and antioxidation, inducing lipid oxidation, DNA damage and ultimately resulting in poor production performance in aged hens [50, 58]. MDA is a significant oxidation product derived from peroxidized polyunsaturated fatty acids and serves as a crucial marker for assessing lipid peroxidation [59]. The findings of our study demonstrated that the incorporation of dietary succinate supplementation led to a significant reduction in MDA and elevated antioxidative enzyme activities, suggesting the suppressed lipid peroxidation. Numerous studies have reported that the antioxidant defense system, which includes T-AOC, CAT and GSH-Px, plays a crucial role in maintaining the balance. T-AOC represented the overall antioxidant effects, CAT converts hydrogen peroxide into water and oxygen [60], and GSH-Px scavenges excessive hydroperoxides [61]. A recent study by Dai et al*.* [62] found that reducing the level of MDA and increasing the activities of T-AOC and GSH-Px could enhance the capacity to scavenge free radicals in ovary, consequently alleviating the laying performance. In this work, the scavenging of oxygen free radicals was investigated to determine its impact on reducing the attack on polyunsaturated fatty acids in biofilms and lipid peroxidation. Moreover, the study aimed to assess whether this scavenging activity could alleviate liver damage. Similarly, in a previous study, Jian et al. [63] confirmed that fatty liver syndrome (FLS), when combined with oxidative stress, inhibited anti-oxidase production and increased ROS levels. Furthermore, the inclusion of feed additives possessing antioxidative properties, such as pyrroloquinoline quinone, demonstrated a hindrance in the prevention of metabolic dysfunction-associated FLD [64].

The gut microbiota plays an essential role in regulating lipid metabolism of the host [65]. It has been proved that the imbalance of microbial community is associated with hepatic lipid metabolism of laying hens, and laying hens with fatty liver exhibited alterations in both microbial abundance and the composition of their gut microbiota [17, 66]. Hamid et al. [10] discovered that aged laying hens with non-alcoholic steatohepatitis (NASH) exhibited changes in the composition of their intestinal microbiota. Specifically, there was an increased abundance of *Bacteroidetes* and *Proteobacteria*, but a significant decrease in the abundance of *Proteobacteria*. It was commonly known that *Bacteroides* was associated with NASH [67]. In our study, the gut microbiota of laying hens was altered by treatment with *Prevotella* and succinate. Particularly, the succinate treatment demonstrated a notable increase in microbial diversity in the cecal contents, significantly influencing the beta diversity and resulting in a pronounced clustering effect on the gut microbes. It indicated that the alleviation of fatty liver in laying hens is associated with the diversity of intestinal microbiota. The finding, consistent with previous research on fatty liver in laying hens, demonstrated a reduction in gut microbiota diversity, while also suggesting that the condition of fatty liver could be mitigated by enhancing gut microbial diversity through the administration of probiotics or prebiotics [53, 68]. For example, Xu et al. [68] reported that *L. salivarius* CML352 led to an increase in the beta diversity of cecal microbiota, a reduction in abdominal fat deposition, and an improvement in egg quality among laying hens. Another relevant study indicated that the inclusion of dietary cholamine could potentially decrease egg production by promoting hepatic lipid deposition and reducing abundances of beneficial intestinal bacteria and microfloral biodiversity in laying hens [66].

More interesting, succinate administration could significantly augment the abundance of *Prevotella* at the genus level, whereas the two strains of *Prevotella* failed to achieve a similar effect. This may be attributed to the relatively mature colonization of intestinal microorganisms in laying hens, and *Prevotella* employed both its own components and metabolites to act on the intestinal flora after entering the body. Upon succinate entering the body, succinate not only exerted direct effect but also significantly promoted the growth of *Prevotella* in the intestinal tract, while synergistically regulating the fat metabolism of laying hens. Similarly, we observed a positive correlation between plasma TG levels and cecal *Prevotella* abundance in this work, providing further evidence for its potential role in alleviating fatty liver in laying hens.

## Conclusions

In summary, this study showed that dietary supplementation of PM and *P. copri* both has a favorable effect on laying performance of hens by reducing hepatic lipid accumulation, suggesting that different species of *Prevotella* has comparable beneficial effect on laying performance and prevention of fatty liver development. The result suggests that the beneficial effect is associated with the manipulation of gut microbiota composition. Dietary supplementation of succinate, the primary metabolite of *Prevotella*, induces a moderate suppression of lipid droplets accumulation in hepatocytes, compared with *Prevotella* treatment. The result highlights that succinate presents a more feasible feed additive for alleviating fatty liver in laying hens. The adequate dietary supplemental level of succinate is warranted to further evaluate the effect of succinate on laying performance and the incidence of fatty liver in laying hen.

### Supplementary Information

**Additional file 1: Fig. S1. **The initial plasma parameters during grouping of hens.

**Additional file 2: Table S1.** Effect of *Prevotella* and sodium succinate on the egg quality at week 4. **Table S2.** Effect of *Prevotella* and sodium succinate on the egg quality at week 8.

## Data Availability

All data generated or analyzed during this study are included in this published article and its supplementary information files.

## Abbreviations

ACC: Acetyl-CoA carboxylase
ASV: Amplicon sequence variant
ATP-5B: Adenosine monophosphate-5B
BA: Bile acid
CAT: Catalase
COX IV: Cytochrome IV
CPT-1: Carnitine palmitoyltransferase 1
FAS: Fatty acid synthase
FFA: Free fat acid
FLD: Fatty liver disease
FLS: Fatty liver syndrome
GLU: Glucose
GSH: Glutathione
GSH-Px: Glutathione peroxidase
H&E: Hematoxylin and eosin
HDL-C: High density lipoprotein cholesterol
HL: Hepatic lipase
IDH3α: Isocitrate dehydrogenase 3α
LDL-C: Low density lipoprotein cholesterol
LEfSe: Linear discriminant analysis effect size
LPL: Lipoprotein lipase
MDA: Malondialdehyde
ME: Malic enzyme
NAFLD: Non-alcoholic fatty liver disease
NASH: Non-alcoholic steatohepatitis
NC: Negative control
NRF1: Nuclear respiratory factor 1
ORO: Oil Red O
OTU: Operational taxonomic unit
*P. Copri*: *Prevotella copri*
PC: Positive control
PCoA: Principal coordinate analysis
PGC-1α: Peroxisome proliferator-activated receptor γ-1α
PLS-DA: Partial least squares discriminant analysis
PM: *Prevotella melaninogenica* group
PPARγ: Peroxisome proliferator-activated receptor γ
RDA: Redundancy analysis
ROS: Reactive oxygen species
SCFAs: Short-chain fatty acids
SOD: Superoxide dismutase
SREBP-1c: Sterol regulatory element binding proteins-1c
Succinate: Succinate group
T-AOC: Total antioxidant capacity
TCH: Total cholesterol
TFAM: Mitochondrial transcription factor A
TG: Triglyceride

## References

[1] Aziza A, Awadin W, Cherian G (2019). Impact of choline supplementation on hepatic histopathology, phospholipid content, and tocopherol status in layer hens fed flaxseed. J Appl Poultry Res.

[2] Davis JE, Cain J, Small C, Hales DB (2016). Therapeutic effect of flax-based diets on fatty liver in aged laying hens. Poult Sci.

[3] Han GP, Kim DY, Kim K, Kim JH, Kil DY (2023). Effect of dietary concentrations of metabolizable energy and neutral detergent fiber on productive performance, egg quality, fatty liver incidence, and hepatic fatty acid metabolism in aged laying hens. Poult Sci.

[4] Song J, Huang M, Shi X, Li X, Chen X, He Z (2021). T329S mutation in the *FMO3* gene alleviates lipid metabolic diseases in chickens in the late laying period. Animals.

[5] Song J, Shi X, Li X, Liang Q, Zeng L, Li G, et al. Associations of the T329S polymorphism in flavin-containing monooxygenase 3 with atherosclerosis and fatty liver syndrome in 90-week-old hens. Front Vet Sci. 2022;9:868602. 10.3389/fvets.2022.868602. 10.3389/fvets.2022.868602 PMC9009339 35433899

[6] Bölükbaşı C, Ürüşan H, Yıldırım BA. The effect of propolis addition into laying hen diet on performance, serum lipid profile and liver fat rate. 2022;Preprint. 10.21203/rs.3.rs-1792056/v1. 10.5194/aab-66-225-2023 PMC10539770 37779600

[7] Chen F, Zhang H, Zhao N, Du E, Jin F, Fan Q, et al. Effects of magnolol and honokiol blend on performance, egg quality, hepatic lipid metabolism, and intestinal morphology of hens at late laying cycle. Animal. 2022;16:100532. 10.1016/j.animal.2022.100532. 10.1016/j.animal.2022.100532 35576638

[8] Mei W, Feng Y, Yao Z, Luo H, Ni Y, Zhao R. Probiotics supplementation alleviate corticosterone-induced fatty liver disease related with the changes of hepatic lipogenesis and gut microbiota profile in broiler. 2020;Preprint. 10.21203/rs.3.rs-35344/v1.

[9] Wieland A, Frank DN, Harnke B, Bambha K (2015). Systematic review: microbial dysbiosis and nonalcoholic fatty liver disease. Aliment Pharmacol Ther.

[10] Hamid H, Zhang JY, Li WX, Liu C, Li ML, Zhao LH (2019). Interactions between the cecal microbiota and non-alcoholic steatohepatitis using laying hens as the model. Poult Sci.

[11] Xiao G, Zheng L, Yan X, Gong L, Yang Y, Qi Q (2022). Effects of dietary essential oils supplementation on egg quality, biochemical parameters, and gut microbiota of late-laying hens. Animals.

[12] Lee NY, Yoon SJ, Han DH, Gupta H, Youn GS, Shin MJ (2020). *Lactobacillus* and *Pediococcus* ameliorate progression of non-alcoholic fatty liver disease through modulation of the gut microbiome. Gut Microbes.

[13] Alaqil AA, Abbas AO, El-Beltagi HS, El-Atty HKA, Mehaisen GMK, Moustafa ES (2020). Dietary supplementation of probiotic *Lactobacillus acidophilus* modulates cholesterol levels, immune response, and productive performance of laying hens. Animals (Basel).

[14] Saleh AA, Eid YZ, Ebeid TA, Ohtsuka A, Hioki K, Yamamoto M (2012). The modification of the muscle fatty acid profile by dietary supplementation with *Aspergillus awamori* in broiler chickens. Br J Nutr.

[15] Bibbò S, Ianiro G, Dore MP, Simonelli C, Newton EE, Cammarota G (2018). Gut microbiota as a driver of inflammation in nonalcoholic fatty liver disease. Mediators Inflamm.

[16] Khogali MK, Wen K, Jauregui D, Malik HEE, Liu L, Zhao M (2022). Probiotics-induced changes in intestinal structure and gut microbiota are associated with reduced rate of pimpled eggs in the late laying period of hens. J Poult Sci.

[17] Liu X, Pan Y, Shen Y, Liu H, Zhao X, Li J, et al. Protective effects of *Abrus cantoniensis* hance on the fatty liver hemorrhagic syndrome in laying hens based on liver metabolomics and gut microbiota. Front Vet Sci. 2022;9:862006. 10.3389/fvets.2022.862006. 10.3389/fvets.2022.862006 PMC9051509 35498747

[18] Sun M, Wu W, Chen L, Yang W, Huang X, Ma C (2018). Microbiota-derived short-chain fatty acids promote Th1 cell IL-10 production to maintain intestinal homeostasis. Nat Commun.

[19] Zhou H, Guo Y, Liu Z, Wu H, Zhao J, Cao Z (2022). Comfrey polysaccharides modulate the gut microbiota and its metabolites SCFAs and affect the production performance of laying hens. Int J Biol Macromol.

[20] Rao Y, Kuang Z, Li C, Guo S, Xu Y, Zhao D, et al. Gut *Akkermansia muciniphila* ameliorates metabolic dysfunction-associated fatty liver disease by regulating the metabolism of L-aspartate via gut-liver axis. Gut Microbes. 2021;13:1927633. 10.1080/19490976.2021.1927633. 10.1080/19490976.2021.1927633 PMC8158032 34030573

[21] Zhu L, Liao R, Huang J, Xiao C, Yang Y, Wang H (2022). *Lactobacillus salivarius* SNK-6 regulates liver lipid metabolism partly via the miR-130a-5p/MBOAT2 pathway in a NAFLD model of laying hens. Cells.

[22] Zhang JL, Xie QM, Ji J, Yang WH, Wu YB, Li C (2012). Different combinations of probiotics improve the production performance, egg quality, and immune response of layer hens. Poult Sci.

[23] Xiang Q, Wang C, Zhang H, Lai W, Wei H, Peng J (2019). Effects of different probiotics on laying performance, egg quality, oxidative status, and gut health in laying hens. Animals (Basel).

[24] De Filippo C, Cavalieri D, Di Paola M, Ramazzotti M, Poullet JB, Massart S (2010). Impact of diet in shaping gut microbiota revealed by a comparative study in children from Europe and rural Africa. P Natl A Sci.

[25] Koeth RA, Wang Z, Levison BS, Buffa JA, Org E, Sheehy BT (2013). Intestinal microbiota metabolism of L-carnitine, a nutrient in red meat, promotes atherosclerosis. Nat Med.

[26] Jiang S, Hu JY, Cheng HW (2022). The impact of probiotic *Bacillus subtilis* on injurious behavior in laying hens. Animals.

[27] Kovatcheva-Datchary P, Nilsson A, Akrami R, Lee YS, De Vadder F, Arora T (2015). Dietary fiber-induced improvement in glucose metabolism is associated with increased abundance of *Prevotella*. Cell Metab.

[28] Péan N, Le Lay A, Brial F, Wasserscheid J, Rouch C, Vincent Mn, et al. Dominant gut *Prevotella copri* in gastrectomised non-obese diabetic Goto-Kakizaki rats improves glucose homeostasis through enhanced FXR signalling. Diabetologia. 2020;63:1223–35. 10.1007/s00125-020-05122-7. 10.1007/s00125-020-05122-7 PMC7228998 32173762

[29] Han H, Yi B, Zhong R, Wang M, Zhang S, Ma J, et al. From gut microbiota to host appetite: gut microbiota-derived metabolites as key regulators. Microbiome. 2021;9:162. 10.1186/s40168-021-01093-y. 10.1186/s40168-021-01093-y PMC8293578 34284827

[30] Barbier-Torres L, Fortner KA, Iruzubieta P, Delgado TC, Giddings E, Chen Y, et al. Silencing hepatic MCJ attenuates non-alcoholic fatty liver disease (NAFLD) by increasing mitochondrial fatty acid oxidation. Nat Commun. 2020;11:3360. 10.1038/s41467-020-16991-2. 10.1038/s41467-020-16991-2 PMC7334216 32620763

[31] Grossini E, Garhwal DP, Calamita G, Romito R, Rigamonti C, Minisini R (2021). Exposure to plasma from non-alcoholic fatty liver disease patients affects hepatocyte viability, generates mitochondrial dysfunction, and modulates pathways involved in fat accumulation and inflammation. Front Med.

[32] Zhao W, Bian Y, Wang Q, Yin F, Yin L, Zhang Y (2022). Blueberry-derived exosomes-like nanoparticles ameliorate nonalcoholic fatty liver disease by attenuating mitochondrial oxidative stress. Acta Pharmacol Sin.

[33] Close B, Banister K, Baumans V, Bernoth EM, Bromage N, Bunyan J, et al. Recommendations for euthanasia of experimental animals: Part 2. DGXT of the European Commission. Lab Anim. 1997;31:1–32. 10.1258/002367797780600297. 10.1258/002367797780600297 9121105

[34] Huang C, Jiao H, Song Z, Zhao J, Wang X, Lin H (2015). Heat stress impairs mitochondria functions and induces oxidative injury in broiler chickens. J Anim Sci.

[35] Trautmann A, Schleicher L, Koch A, Günther J, Steuber J, Seifert J. A shift towards succinate-producing *Prevotella* in the ruminal microbiome challenged with monensin. Proteomics. 2022;23:e2200121. 10.1002/pmic.202200121. 10.1002/pmic.202200121 36444514

[36] Gan L, Zhao Y, Mahmood T, Guo Y (2020). Effects of dietary vitamins supplementation level on the production performance and intestinal microbiota of aged laying hens. Poult Sci.

[37] Zhu L, Liao R, Wu N, Zhu G, Yang C (2019). Heat stress mediates changes in fecal microbiome and functional pathways of laying hens. Appl Microbiol Biotechnol.

[38] Wang F, Zou P, Xu S, Wang Q, Zhou Y, Li X, et al. Dietary supplementation of *Macleaya cordata* extract and *Bacillus* in combination improve laying performance by regulating reproductive hormones, intestinal microbiota and barrier function of laying hens. J Anim Sci Biotechnol. 2022;13:118. 10.1186/s40104-022-00766-4. 10.1186/s40104-022-00766-4 PMC9559840 36224643

[39] Neijat M, Shirley RB, Barton J, Thiery P, Welsher A, Kiarie E (2019). Effect of dietary supplementation of *Bacillus subtilis* DSM29784 on hen performance, egg quality indices, and apparent retention of dietary components in laying hens from 19 to 48 weeks of age. Poult Sci.

[40] Guo L, Kuang J, Zhuang Y, Jiang J, Shi Y, Huang C, et al. Serum metabolomic profiling to reveal potential biomarkers for the diagnosis of fatty liver hemorrhagic syndrome in laying hens. Front Physiol. 2021;12:590638. 10.3389/fphys.2021.590638. 10.3389/fphys.2021.590638 PMC7900428 33633583

[41] Whitehead C (1979). Nutritional and metabolic aspects of fatty liver disease in poultry. Vet Q.

[42] Yang F, Ruan J, Wang T, Luo J, Cao H, Song Y (2017). Improving effect of dietary soybean phospholipids supplement on hepatic and serum indexes relevant to fatty liver hemorrhagic syndrome in laying hens. Anim Sci J.

[43] Wu X, Zou X, Zhang M, Hu H, Wei X, Jin M (2021). Osteocalcin prevents insulin resistance, hepatic inflammation, and activates autophagy associated with high-fat diet-induced fatty liver hemorrhagic syndrome in aged laying hens. Poult Sci.

[44] Zhu Y, Zhang X, Du P, Wang Z, Luo P, Huang Y, et al. Dietary herbaceous mixture supplementation reduced hepatic lipid deposition and improved hepatic health status in post-peak laying hens. Poult Sci. 2022;101:101870. 10.1016/j.psj.2022.101870. 10.1016/j.psj.2022.101870 PMC9061633 35472740

[45] Jin J, Cheng R, Ren Y, Shen X, Wang J, Xue Y (2021). Distinctive gut microbiota in patients with overweight and obesity with dyslipidemia and its responses to long-term orlistat and ezetimibe intervention: a randomized controlled open-label trial. Front Pharmacol.

[46] Liao Q, Wu T, Fu Q, Wang P, Zhao Y, Li Y (2022). Effects of dietary inclusion of β-hydroxy-β-methylbutyrate on growth performance, fat deposition, bile acid metabolism, and gut microbiota function in high-fat and high-cholesterol diet-challenged layer chickens. Curr Issues Mol Biol.

[47] Yao H, Hu Y, Wang Q, Zhang Y, Rao K, Shi S. Effects of dietary dimethylglycine supplementation on laying performance, egg quality, and tissue index of hens during late laying period. Poult Sci. 2022;101:101610. 10.1016/j.psj.2021.101610. 10.1016/j.psj.2021.101610 PMC8704446 34936951

[48] Song Y, Ruan J, Luo J, Wang T, Yang F, Cao H (2017). Abnormal histopathology, fat percent and hepatic apolipoprotein A I and apolipoprotein B100 mRNA expression in fatty liver hemorrhagic syndrome and their improvement by soybean lecithin. Poult Sci.

[49] Mack KD, Walzem RL, Lehmann-Bruinsma K, Powell JS, Zeldis JB (1996). Polylysine enhances cationic liposome-mediated transfection of the hepatoblastoma cell line Hep G2. Biotechnol Appl Biochem.

[50] Lv Z, Xing K, Li G, Liu D, Guo Y (2018). Dietary genistein alleviates lipid metabolism disorder and inflammatory response in laying hens with fatty liver syndrome. Front Physiol.

[51] Rozenboim I, Mahato J, Cohen N, Tirosh O (2016). Low protein and high-energy diet: a possible natural cause of fatty liver hemorrhagic syndrome in caged White Leghorn laying hens. Poult Sci.

[52] Wei F, Yang X, Zhang M, Xu C, Hu Y, Liu D. *Akkermansia muciniphila* enhances egg quality and the lipid profile of egg yolk by improving lipid metabolism. Front Microbiol. 2022;13:927245. 10.3389/fmicb.2022.927245. 10.3389/fmicb.2022.927245 PMC9344071 35928144

[53] Wang W, Wang J, Zhang H, Wu S, Qi G (2020). Supplemental *Clostridium butyricum* modulates lipid metabolism through shaping gut microbiota and bile acid profile of aged laying hens. Front Microbiol.

[54] Donnelly KL, Smith CI, Schwarzenberg SJ, Jessurun J, Boldt MD, Parks EJ (2005). Sources of fatty acids stored in liver and secreted via lipoproteins in patients with nonalcoholic fatty liver disease. J Clin Invest.

[55] Liu CY, Grant AL, Kim KH, Mills SE (1994). Porcine somatotropin decreases acetyl-CoA carboxylase gene expression in porcine adipose tissue. Domest Anim Endocrinol.

[56] Postic C, Girard J (2008). Contribution of de novo fatty acid synthesis to hepatic steatosis and insulin resistance: lessons from genetically engineered mice. J Clin Invest.

[57] Hu Y, Sun Q, Hu Y, Hou Z, Zong Y, Omer NA (2018). Corticosterone-induced lipogenesis activation and lipophagy inhibition in chicken liver are alleviated by maternal betaine supplementation. J Nutr.

[58] Seo E, Kang H, Choi H, Choi W, Jun HS. Reactive oxygen species-induced changes in glucose and lipid metabolism contribute to the accumulation of cholesterol in the liver during aging. Aging Cell. 2019;18:e12895. 10.1111/acel.12895. 10.1111/acel.12895 PMC6413652 30609251

[59] Freeman BA, Crapo JD (1981). Hyperoxia increases oxygen radical production in rat lungs and lung mitochondria. J Biol Chem.

[60] Biazus AH, Da Silva AS, Bottari NB, Baldissera MD, do Carmo GM, Morsch VM, et al. Fowl typhoid in laying hens cause hepatic oxidative stress. Microb Pathog. 2017;103:162–6. 10.1016/j.micpath.2016.12.009. 10.1016/j.micpath.2016.12.009 28027943

[61] Gümüş P, Emingil G, Öztürk V-Ö, Belibasakis GN, Bostanci N. Oxidative stress markers in saliva and periodontal disease status: modulation during pregnancy and postpartum. BMC Infect Dis. 2015;15:261. 10.1186/s12879-015-1003-z. 10.1186/s12879-015-1003-z PMC4495776 26152310

[62] Dai H, Lv Z, Huang Z, Ye N, Li S, Jiang J, et al. Dietary hawthorn-leaves flavonoids improves ovarian function and liver lipid metabolism in aged breeder hens. Poult Sci. 2021;100:101499. 10.1016/j.psj.2021.101499. 10.1016/j.psj.2021.101499 PMC8572884 34731736

[63] Jian H, Xu Q, Wang X, Liu Y, Miao S, Li Y, et al. Amino acid and fatty acid metabolism disorders trigger oxidative stress and inflammatory response in excessive dietary valine-induced NAFLD of laying hens. Front Nutr. 2022;9:849767. 10.3389/fnut.2022.849767. 10.3389/fnut.2022.849767 PMC9040670 35495903

[64] Qiu K, Zhao Q, Wang J, Qi G-H, Wu S-G, Zhang H-J (2021). Effects of pyrroloquinoline quinone on lipid metabolism and anti-oxidative capacity in a high-fat-diet metabolic dysfunction-associated fatty liver disease chick model. Int J Mol Sci.

[65] Ushiroda C, Naito Y, Takagi T, Uchiyama K, Mizushima K, Higashimura Y (2019). Green tea polyphenol (epigallocatechin-3-gallate) improves gut dysbiosis and serum bile acids dysregulation in high-fat diet-fed mice. J Clin Biochem Nutr.

[66] Wu G, Li Z, Zheng Y, Zhang Y, Liu L, Gong D, et al. Supplementing cholamine to diet lowers laying rate by promoting liver fat deposition and altering intestinal microflora in laying hens. Poult Sci. 2022;101:102084. 10.1016/j.psj.2022.102084. 10.1016/j.psj.2022.102084 PMC9449860 36055021

[67] Boursier J, Diehl AM (2016). Nonalcoholic fatty liver disease and the gut microbiome. Clin Liver Dis.

[68] Xu C, Wei F, Yang X, Feng Y, Liu D, Hu Y (2022). *Lactobacillus salivarius* CML352 isolated from Chinese local breed chicken modulates the gut microbiota and improves intestinal health and egg quality in late-phase laying hens. Microorganisms.

