# Supplementary material for: Prevotella and succinate treatments altered gut microbiota, increased laying performance, and suppressed hepatic lipid accumulation in laying hens
Source: J Anim Sci Biotechnol. 2024 Feb 18;15:26. doi: 10.1186/s40104-023-00975-5 (PMC10874536; doi:10.1186/s40104-023-00975-5)
Supplement: Supplementary file 1 — Additional file 1: Fig. S1. The initial plasma parameters during grouping of hens. [file 40104_2023_975_MOESM1_ESM.docx]

**Fig. S1** The initial plasma parameters during grouping of hens. **A** Triglyceride (TG). **B** Total cholesterol (TCH). **C** Glucose (GLU). **D** Low-density lipoprotein-cholesterol (LDL-C). **E** High-density lipoprotein-cholesterol (HDL-C)
