# Supplementary material for: Prevotella and succinate treatments altered gut microbiota, increased laying performance, and suppressed hepatic lipid accumulation in laying hens
Source: J Anim Sci Biotechnol. 2024 Feb 18;15:26. doi: 10.1186/s40104-023-00975-5 (PMC10874536; doi:10.1186/s40104-023-00975-5)
Supplement: Supplementary file 2 — Additional file 2: Table S1. Effect of Prevotella and sodium succinate on the egg quality at week 4. Table S2. Effect of Prevotella and sodium succinate on the egg quality at week 8. [file 40104_2023_975_MOESM2_ESM.docx]

**Table S1** Effect of *Prevotella* and sodium succinate on the egg quality at week 4

|  | NC | PC | PM | P. copri | Succinate | *P* value |
| --- | --- | --- | --- | --- | --- | --- |
| Egg-shaped index | 1.32±0.01 | 1.27±0.06 | 1.30±0.01 | 1.30±0.01 | 1.29±0.01 | 0.765 |
| Eggshell thickness, mm | 0.32±0.01 | 0.31±0.01 | 0.31±0.01 | 0.31±0.01 | 0.32±0.01 | 0.631 |
| Eggshell strength, kg·f | 3.29±0.36 | 3.08±0.30 | 3.06±0.19 | 3.00±0.24 | 3.71±0.25 | 0.327 |
| Albumen height, mm | 5.15±0.64 | 5.54±0.84 | 5.61±0.60 | 4.81±0.74 | 6.18±0.91 | 0.760 |
| Yolk color | 4.42±0.24 | 4.18±0.20 | 4.33±0.27 | 4.30±0.21 | 4.48±0.18 | 0.897 |
| Haugh units | 67.44±6.07 | 69.82±6.60 | 70.29±4.95 | 61.97±5.65 | 74.76±6.89 | 0.673 |
| Yolk proportion, % | 27.92±0.76 | 28.24±0.52 | 27.43±0.70 | 28.59±0.38 | 28.44±0.52 | 0.667 |
| Eggshell proportion, % | 10.14±0.46 | 9.82±0.48 | 10.03±0.20 | 10.14±0.23 | 10.40±0.46 | 0.874 |

**Table S2** Effect of *Prevotella* and sodium succinate on the egg quality at week 8

|  | NC | PC | PM | P. copri | Succinate | *P* value |
| --- | --- | --- | --- | --- | --- | --- |
| Shape index, % | 1.30±0.93 | 1.33±0.01 | 1.29±0.03 | 1.33±0.01 | 1.34±0.01 | 0.530 |
| Shell thickness, mm | 0.32±0.01 | 0.30±0.01 | 0.32±0.01 | 0.32±0.01 | 0.32±0.01 | 0.530 |
| Breaking strength, kg·f | 3.43±0.42 | 3.65±0.32 | 3.60±0.15 | 3.50±0.25 | 3.94±0.26 | 0.783 |
| Eggshell percentage, % | 9.94±0.27 | 9.97±0.61 | 10.07±0.19 | 10.60±0.24 | 10.48±0.29 | 0.313 |
